# Association of the hemoglobin, albumin, lymphocyte, and platelet score with the risk of Erectile dysfunction: a cross-sectional study

**DOI:** 10.1038/s41598-024-66667-w

**Published:** 2024-07-09

**Authors:** Di Chen, Jinji Chen, Qiufeng Zhou, Hua Mi, Gang Liu

**Affiliations:** 1 The Department of Urology, Reproductive Hospital of Guangxi Zhuang Autonomous Region, Nanning, China; 2 The Department of Urology, The First Affiliated Hospital of Guangxi Medical University, Nanning, China; 3 The 924th Hospital of Chinese PLA Joint Service Support Force, Guilin, China

**Keywords:** HALP, Erectile dysfunction, National Health and Hutrition Hxamination Survey, Platelet, Hemoglobin, Lymphocyte, Albumin, Sexual dysfunction, Diagnostic markers

## Abstract

Erectile dysfunction (ED) is related to nutritional and inflammatory factors. The hemoglobin, albumin, lymphocyte, and platelet score (HALP), a new index reflecting the nutritional and inflammatory status, has been associated with a higher risk of diabetic retinopathy, particularly at lower values (≤ 42.9). However, studies focusing on the relationship between HALP and ED risk are scarce. Hence, this study aimed to investigate the association between HALP and ED. Data were extracted from the National Health and Nutrition Examination Survey (NHANES) conducted between 2001 and 2004. Based on self-reported data, participants were classified into either the ED group or the non-ED group. Next, the HALP score was categorized into four quartiles (Q1–4). Weighted multivariate regression analysis was performed to assess the relationship between categorical HALP and ED risk. Additionally, restricted cubic spline (RCS) analysis was conducted to examine the association between continuous HALP scores and ED risk. Furthermore, subgroup analyses were conducted to examine the association between categorical HALP and the risk of ED based on age, as well as the status of hypertension, diabetes and cardiovascular disease. Finally, a mediation analysis was carried out to investigate the mediating effect of HALP and related parameters on the association between urinary cobalt levels and ED. Initially, the data of 21,161 participants were collected. After implementing the inclusion and exclusion criteria, 3406 participants were included in the final analyses. Weighted multivariate regression analysis demonstrated that the Q4 HALP group was associated with a lower risk of ED (OR 0.96, 95% confidence intervals 0.92–1.00, P = 0.037). Meanwhile, RCS analysis showed that HALP was nonlinearly associated with the risk of ED. In addition, subgroup analyses demonstrated that participants in the Q3/4 HALP group had a significantly lower ED risk than those in the Q1 group among patients aged ≥ 50 years, as well as those with hypertension and diabetes. Lastly, mediation analysis revealed that HALP and its associated parameters had a marginal average causal mediation effect on the relationship between urinary cobalt levels and ED risk (P > 0.05). In US adults, high HALP scores were correlated with a lower risk of ED. The relationship was more pronounced in participants aged ≥ 50 years with hypertension and diabetes. Furthermore, HALP and its parameters may not mediate the association between urinary cobalt levels and ED risk.

## Introduction

Erectile dysfunction (ED) is a prevalent male sexual dysfunction defined as the persistent inability to attain or maintain a penile erection sufficient for satisfactory sexual performance^1^. Approximately 36% of men aged over 40 years suffer from ED^2^. As is well documented, its incidence has been on the rise among men younger than 40 years. According to a large-scale transnational study, the incidence of ED in young men was as high as 30%^3^. In Britain, a prospective real-world study involving 12,490 men documented that 41.5% of British men manifested signs of ED, among which 7.5% experienced severe ED^4^. ED is a multifactorial condition affected by cavernosal, hormonal, vascular, neurogenic, and drug-induced factors. Advanced age, obesity, and a history of smoking, depression, hypertension, and diabetes have been identified as risk factors for the development of ED. Existing evidence suggests that systemic inflammatory conditions lead to endothelial dysfunction, which is associated with several vascular diseases^5^. Due to the smaller size and limited tolerance of penile arteries, ED may be an early indicator of systemic endothelial dysfunction^6^. Thus, the relationship between inflammation and ED has recently garnered extensive attention.

Inflammation is a physiological response that protects organisms from infections or tissue injury. However, dysregulation of immune response and inflammation contributes to the pathogenesis of various diseases. Several immune-inflammatory markers, such as lymphocytes count, neutrophils count, neutrophil-to-lymphocyte ratio (NLR), platelet-to-lymphocyte ratio (PLR), and C-reactive protein (CRP) levels, are commonly used to evaluated the status of systemic inflammation and have been reported to be associated with the development of ED. Liu et al. performed a meta-analysis involving 12 studies and found higher CRP levels in ED patients compared to healthy controls. Moreover, they described that PDE5I therapy can restore CRP levels^7^. In 2015, Chen et al. proposed a prognostic score based on preoperative hemoglobin and albumin levels, as well as lymphocyte and platelet counts, and concluded that gastric cancer patients with a score higher than 56.8 had a more favorable prognosis^8^. In additions, hemoglobin, albumin, lymphocyte, and platelet (HALP) scores showed superior predictive value than TNM staging alone in these gastric cancer patients^8^. HALP score, as a new integrated indicator that evaluates the inflammatory and nutritional status of patients, has been associated with the prognosis of cancer or cardiovascular disease patients^9,10^. Moreover, HALP has also been reported to be associated with the incidence of non-neoplastic diseases. Ding et al. evinced that a lower HALP (≤ 42.9) value was an independent risk factor for diabetic retinopathy^11^. However, to data, studies analyzing the correlation between HALP scores and ED risk are limited. Of note, previous studies indicated that urine heavy metal exposure, such as cobalt and antimony, increases ED risk^12^. As a nutritional and inflammation-related indicator, HALP and its parameters may serve as mediators between urinary cobalt exposure and the risk of ED.

The objective of this study was to explore the association between HALP score and the risk of ED using data derived from the National Health and Nutrition Examination Survey (NHANES). In addition, mediating analyses were performed to investigate the role of HALP and its parameters in mediating the association between urinary cobalt exposure and ED risk.

## Materials and methods

### Data extraction and screening

The NHANES database contains several data modules, including demographics, dietary habits, examinations, laboratory test, and questionnaires. In additions, the studies involving human participants were reviewed and approved by the National Center for Health Statistics (NCHS) Research Ethics Review Committee. All detailed NHANES study designs and data are publicly available at www.cdc.gov/nchs/nhanes/. Upon inclusion, participants were assigned a unique code to facilitate across each module. Furthermore, each item was assigned a unique identifier to ensure consistency across NHANES cycles. Therefore, data from different NHANES cycles can be pooled to increase the sample size.

Given that ED related questionnaire was exclusively available for participants aged 20 years and over between 2001 and 2004, data from the 2001–2002 to 2003–2004 NHANES cycles were pooled. Demographic data comprised age, race, educational level, annual household income, and marital status. Body mass index was calculated from the examination records. Laboratory data included complete blood cell count, as well as albumin and urine cobalt levels. Information on smoking status, alcohol consumption, physical activity levels, status of diabetes, hypertension, cardiovascular disease, and ED were obtained from the questionnaire module. The inclusion criteria were as follows: (1) Available ED data; (2) available data on hemoglobin level, albumin level, lymphocyte count, and platelet count. The exclusion criteria were as follows: (1) Missing or incomplete data on educational level, marital status, annual household income, smoking status, alcohol consumption, and physical activity; (2) Missing or incomplete data on hypertension, diabetes, and cardiovascular disease; (3) participants diagnosed with prostate cancer.

### Outcome and exposure variables

The exposure variable was the HALP score, which was calculated using the following formula: hemoglobin level (g/L) × albumin level (g/L) × lymphocytes count (/L)/platelets count (/L). The code number for lymphocytes, hemoglobin, and platelets are “LBDLYMNO”, “LBXHGB”, and “LBXPLTSI” in the complete blood count item, respectively. The code number for albumin is “LBDSALSI” in the standard biochemistry item. Venous blood samples were collected at the mobile examination center (MEC). The Beckman Coulter MAXM was used to measure blood cell count between 2001 and 2004, whereas the Beckman Synchron LX20 was employed to detect serum albumin levels between 2001 and 2004.

The outcome variable was ED. In the questionnaire data module, participants were asked about their ability to maintain an erection. If they responded “Always or almost always able” and “Usually able”, they were assigned to the non-ED group. If participants responded “Sometimes able” and “Never able”, they were assigned to the ED group.

### Covariates

Besides outcome and exposure variables, the remaining variables served as covariates. Some covariates were treated as categorical variables. Age was categorized into the < 50 years and 50 years and above subgroups^5^. Annual family income was categorized into the < $ 20,000 and ≥ $20,000 subgroups^13^. Body mass index was categorized into < 25, 25–29.99, and ≥ 30 kg/m^2^ subgroups. The remaining categorical covariates were defined as follows: Educational level was categorized into less than high school, high school, and college or above. Marital status is dichotomized into married or living with a partner, and living alone. Regarding cigarette smoking status, participants were classified as no (smoked fewer than 100 cigarettes in a lifetime) and yes (smoked at least 100 cigarettes in a lifetime). Alcohol intake was classified as no (drinking fewer than 12 alcoholic drinks per year) and yes (drinking at least 12 alcoholic drinks per year). History of diabetes, hypertension, and cardiovascular disease were dichotomized as yes and no according to responses to the questionnaires. Physical activity was further categorized into moderate or vigorous physical activity according to the questionnaire “Vigorous activity and moderate activity over the past 30 days”. Furthermore, cardiovascular disease included coronary heart disease, angina, and stroke. Other covariates were categorized based on NHANES categorization criteria.

### Statistical analysis

Analyses were performed using survey methods tailored for complex sampling designs with appropriate strata, primary sampling unit, and sampling weights. Given that some variables were measured at the MEC, MEC examination weights recorded “WTMEC2YR” were applied to all analyses. Continuous variables were expressed as means and 95% confidence intervals (CI), whereas categorical variables were represented as the number of cases (percentage). Group comparisons were performed using the Wilcoxon rank-sum test and chi-square test for complex survey samples. HALP score were categorized into four quartiles: 25% (Quartile 1, Q1), 50% (Quartile 2, Q2), 75% (Quartile 3, Q3), and 100% (Quartile 4, Q4), with Q1 and Q4 representing the lowest and highest HALP levels, respectively.

Weighted multivariable logistic regression was utilized to analyze the association between HALP and the risk of ED in three models. Model 1 unadjusted for covariates, Model 2 was adjusted for age, race, educational level, marital status, annual family income, and body mass index, and Model 3 was adjusted for all covariates. Additionally, restricted cubic spline (RCS) analysis was carried out to explore the potential association between continuous HALP and the odd ratio of ED. Subgroup analysis stratified by age, hypertension, diabetes, and cardiovascular disease was performed to examine the association between HALP and ED risk in model 3. The interaction effect of HALP-ED relation and these subgroups were assessed. Finally, a mediation analysis between urinary cobalt levels and ED was conducted to determine the mediation effect of HALP and related parameters. Two-tailed P values less than 0.05 were considered statistically significant. Statistical analyses were performed using R software version 4.2.2 (http://www.R-project.or). Primary R packages included “haven”, “survey”, “gtsummary”, and “mediate”.

## Results

### Participant characteristics

**Figure 1:**
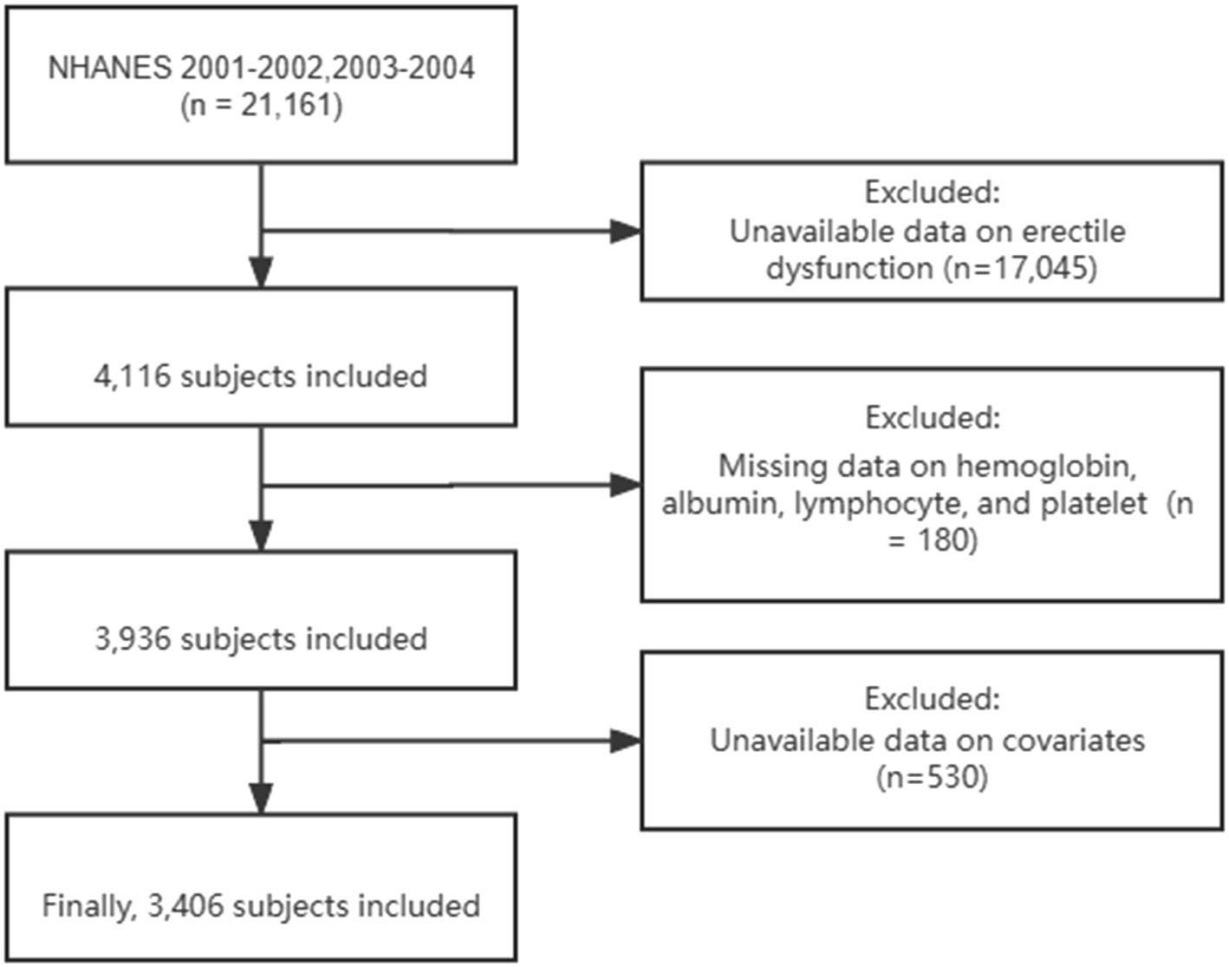
Flow chart selection process.

The clinical information of participants was sourced from the 2001 to 2004 NHANES dataset. After applying the inclusion and exclusion criteria, 3406 participants were included in this study. The specific screening process is illustrated in Fig. 1. Among the 3406 participants, 906 self-reported experiencing abnormal erectile function. As summarized in Table 1, approximately 80% of participants were ≥ 50 years old, 29% had an educational level less than high school, 19% had an annual family income under $20,000, 36% had a body mass index ≥ 30 kg/m^2^, 70% were smokers, 49% suffered from hypertension, and 21% had diabetes in the ED group. Continuous HALP was significantly lower in participants with ED than in those without ED (49.45 vs 53.72, P = 0.002).

**Table 1:** Baseline characteristics of participants.

Characteristic	Erectile dysfunction		P-value^1^
	No (N = 2500)	Yes (N = 906)	
Age (years)			< 0.001
< 50	1690 (74%)	130 (20%)	
≥ 50	810 (26%)	776 (80%)	
Race			0.3
Non-Hispanic White	1354 (75%)	537 (77%)	
Mexican American	497 (7.6%)	185 (6.8%)	
Non-Hispanic Black	485 (9.4%)	134 (8.2%)	
Other Hispanic	82 (4.1%)	33 (5.2%)	
Other race—including multi-racial	82 (4.1%)	17 (2.7%)	
Marital status			< 0.001
Married or living with partner	1712 (70%)	677 (78%)	
Living alone	788 (30%)	229 (22%)	
Education level			< 0.001
Above high school	1289 (59%)	365 (48%)	
Less than high school	553 (13%)	361 (29%)	
High school or GED	658 (28%)	180 (23%)	
Annual family income			< 0.001
$20,000 and over	2081 (88%)	657 (81%)	
Under $20,000	419 (12%)	249 (19%)	
Body mass index (kg/m^2^)			0.002
< 25	760 (30%)	235 (24%)	
25–29.99	1044 (41%)	386 (40%)	
≥ 30	696 (28%)	285 (36%)	
Alcohol intaking			0.010
Yes	2105 (85%)	734 (80%)	
No	395 (15%)	172 (20%)	
Cigarette smoking			< 0.001
Yes	1387 (54%)	645 (70%)	
No	1113 (46%)	261 (30%)	
Physical activity status			
Vigorous			< 0.001
No	1432 (54%)	671 (72%)	
Yes	1028 (45%)	160 (20%)	
Unable to do activity	40 (1.2%)	75 (7.7%)	
Moderate			< 0.001
No	1143 (40%)	468 (46%)	
Yes	1338 (59%)	391 (48%)	
Unable to do activity	19 (0.7%)	47 (5.5%)	
Diabetes			< 0.001
No	2378 (97%)	692 (79%)	
Yes	122 (3.4%)	214 (21%)	
Hypertension			< 0.001
No	1926 (79%)	433 (51%)	
Yes	574 (21%)	473 (49%)	
Cardiovascular disease			< 0.001
No	2372 (96%)	694 (80%)	
Yes	128 (4.0%)	212 (20%)	
HALP (categorical)			0.003
Q1	601 (24%)	304 (32%)	
Q2	618 (25%)	217 (25%)	
Q3	636 (26%)	188 (22%)	
Q4	645 (26%)	197 (21%)	
HALP (continuous)	53.72 (42.08, 68.36)	49.45 (38.79, 64.21)	0.002

*HALP* hemoglobin, albumin, lymphocyte, and platelet.

^1^Chi-squared test with Rao & Scott’s second-order correction; Wilcoxon rank-sum test for complex survey samples.

### The association between HALP and ED

**Figure 2:**
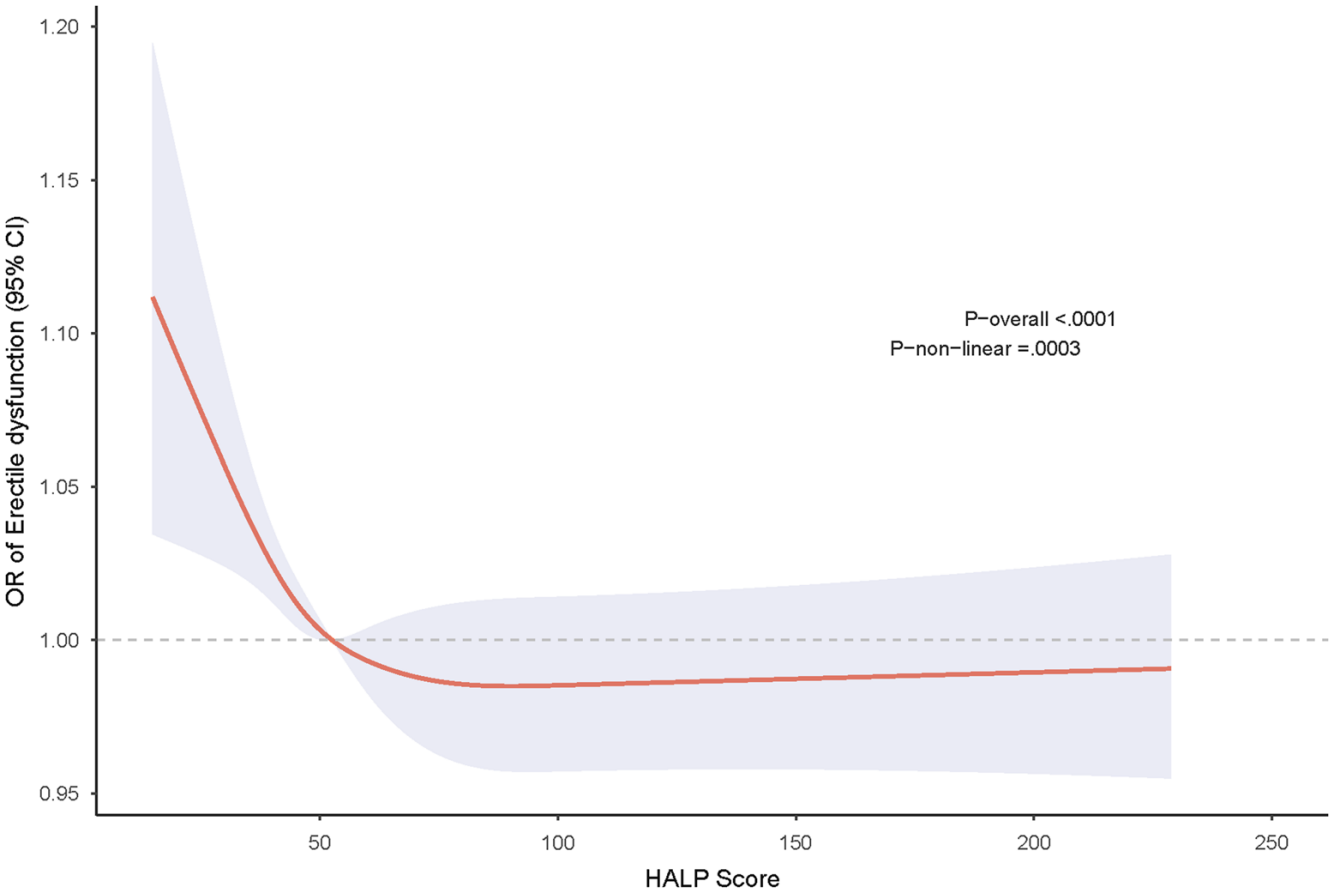
Restricted cubic spline for hemoglobin, albumin, lymphocyte, and platelet score and erectile dysfunction risk.

Weighted multivariable logistic regression was used to examine the relationship between categorical HALP levels and ED risk in each model. The results revealed a significant association between Q4 HALP and lower ED risk. In model 3, the Q4 group had a 4% lower risk of ED compared with the Q1 group (Table 2). In model 1, the OR (95% CI) of ED was 0.92 (0.88–0.97) in the Q4 group compared with the Q1 group. As displayed in Fig. 2, the RCS plot delineated a nonlinear relationship between continuous HALP score and the risk of ED (P-overall < 0.01, P-nonlinear < 0.01).

**Table 2:** Weighted multivariable logistic regression for the association between the HALP and Erectile dysfunction risk.

	OR (95% CI), P-value		
	Model 1	Model 2	Model 3
HALP (categorical)			
Q1	Reference	Reference	Reference
Q2	0.95 (0.92, 0.99), 0.010	0.97 (0.94, 1.00), 0.094	0.98 (0.95, 1.01), 0.11
Q3	0.93 (0.89, 0.98), 0.007	0.97 (0.93, 1.00), 0.077	0.97 (0.94, 1.01), 0.11
Q4	0.92 (0.88, 0.97), 0.002	0.96 (0.92, 1.00), 0.048	0.96 (0.92, 1.00), 0.037

*OR* odds ratio, *CI* confidence interval, *Q1–Q4* quartile 1–quartile 4, *HALP* hemoglobin, albumin, lymphocyte, and platelet.

Model 1: Unadjusted; Model 2: Adjusted for age, race, marital status, education level, annual family income, body mass index; Model 3: Adjusted for Model 2+ alcohol intaking, cigarette smoking, physical activity status, diabetes, hypertension, cardiovascular disease.

### Subgroup analysis

Subgroup analyses were performed to explore variations in the relationship between HALP and ED risk based on participants characteristics. As anticipated, participants in the Q3/4 HALP group had a lower ED risk than participants in the Q1 group in age ≥ 50 years, hypertension, and diabetes subgroups (Table 3). However, covariates such as age, hypertension, and diabetes may influence the relationship between HALP and ED risk (P value for interaction < 0.05).

**Table 3:** Subgroup analysis for the relationship between HALP and erectile dysfunction risk in model 3.

	HALP, OR (95% CI)				P value for interaction^1^
	Q1	Q2	Q3	Q4	
Age (years)					0.031
< 50	Reference	0.99 (0.97, 1.02)	1.01 (0.97, 1.05)	0.99 (0.95, 1.03)	
≥ 50	Reference	0.96 (0.89, 1.02)	0.91 (0.85, 0.98)*	0.91 (0.83, 1.00)*	
Hypertension					0.020
Yes	Reference	0.97 (0.90, 1.05)	0.89 (0.83, 0.97)*	0.90 (0.84, 0.96)*	
No	Reference	0.98 (0.94, 1.01)	1.00 (0.96, 1.03)	0.98 (0.93, 1.02)	
Diabetes					0.008
Yes	Reference	0.88 (0.73, 1.06)	0.78 (0.68, 0.90)*	0.84 (0.70, 1.00)	
No	Reference	0.99 (0.96, 1.02)	0.99 (0.95, 1.02)	0.97 (0.93, 1.00)	
Cardiovascular disease					0.200
Yes	Reference	0.91 (0.78, 1.05)	0.90 (0.77, 1.05)	0.93 (0.77, 1.12)	
No	Reference	0.98 (0.95, 1.01)	0.98 (0.94, 1.01)	0.96 (0.92, 1.00)	

*HALP* hemoglobin, albumin, lymphocyte, and platelet.

^1^Interaction analysis between selected subgroup.

*P-value < 0.05.

### Mediating effect of HALP

Participants with complete urine cobalt levels were included in the analysis. As presented in Table 4, HALP did not mediate the association between urinary cobalt levels and ED risk (average causal mediation effect [ACME] = 0.000253, P = 0.276). Similarly, the four HALP parameters (hemoglobin, albumin, lymphocyte, and platelet) also did not mediate this association (ACME-P > 0.05). Notably, urinary cobalt may directly contribute to an increased risk of ED (average direct effect [ADE] = 0.005991, P = 0.046).

**Table 4:** Mediation analyses between urine cobalt and Erectile dysfunction.

	ACME		ADE		Total effect		Proportion mediated	
	Estimate	P-value	Estimate	P-value	Estimate	P-value	Estimate	P-value
HALP	0.000253	0.276	0.006047	0.054	0.006300	0.056	0.040186	0.252
Albumin	0.00014	0.170	0.00616	0.094	0.006300	0.084	0.02221	0.234
Hemoglobin	0.0000816	0.568	0.00622	0.060	0.006300	0.056	0.0129	0.580
Lymphocyte	0.000309	0.464	0.005991	0.046	0.006300	0.056	0.049049	0.420
Platelet	0.00000472	0.948	0.00630	0.052	0.00630	0.056	0.000749	0.924

*ACME* average causal mediation effect, *ADE* average direct effect, *HALP* hemoglobin, albumin, lymphocyte, and platelet.

## Discussion

This cross-sectional study included 3406 participants, among which 906 participants had ED. Interestingly, an association was observed between high HALP scores and a lower ED risk. In the weighted multivariable logistic regression with all covariate adjusted, the average risk of ED was significantly lower in the Q4 group compared to the Q1, Q2 and Q3 groups. Furthermore, subgroup analyses exposed that HALP was associated with a higher risk of ED in participants aged ≥ 50 years and those with hypertension and diabetes. However, HALP and its individual parameters were not found to mediate the association between urinary cobalt levels and ED risk. While HALP has been established to be associated with tumor prognosis, to the best or our knowledge, this is the first study to analyze the relationship between HALP score and ED risk.

Owing to the combination of nutritional and immune status, HALP is commonly used in the preoperative and prognostic assessment of cancer patients. Of note, HALP has been found to be significantly related to tumor prognosis, metastasis, acute mechanical intestinal obstruction, post-stroke cognitive impairment, and cerebral venous sinus thrombosis. Pan et al. performed a study based on data from the NHANES and determined that the HALP score was non-linearly correlated with all-cause mortality and cardiovascular mortality^10^. Hemoglobin level and platelet count were factors that significantly affected this association. Ekinci et al. performed a retrospective cohort study of 123 metastatic renal cell cancer patients and evinced that patients with high HALP scores had superior overall survival outcomes^14^. Albumin, which reflects the nutritional status, has been associated with the prognosis of renal cell carcinoma^15^. The decrease in lymphocyte count may indicate compromised immune responses to tumors. Meanwhile, platelets can protect tumor cells from natural killer cells^16^. Likewise, Duran et al. performed an observational study that recruited 307 breast cancer patients and reported that low HALP scores were associated with aggressive tumor behaviour^17^. Moreover, the HALP score is a valuable clinical tool for distinguishing between acute mechanical intestinal obstruction due to benign and malignant origins. Akbas et al. implied that HALP scores < 23.94 were associated with malignant intestinal obstruction^18^. Xu et al. investigated 592 individuals with ischemic stroke and unveiled that low HALP scores were correlated with early-onset post-stroke cognitive impairment^19^. Malnutrition and systemic inflammation may account for the low HALP score in patients with poor clinical outcome. At present, reports on the role of HALP in benign diseases are scarce. Our study noted a low risk of ED in participants with high HALP scores.

Low HALP levels can be attributed to a relative reduction in hemoglobin and albumin levels, lymphocyte count, or platelet count. Hemoglobin level is a robust indicator of oxygen transport. Long-term malnutrition and anemia-related diseases can result in a decrease in hemoglobin levels. Chen et al. performed a retrospective cohort study and noted that elderly patients with thalassemia had a higher risk of developing ED risk than young, healthy men post-blood transfusion. Hence, anemia may be a contributing factor to ED. Albumin, a by-product of the liver, is closely related to nutritional status. Nevertheless, limited studies reported the role of albumin in ED. Toda et al. performed a comparative study and identified albumin as an independent risk factor for ED. In healthy subjects, albumin-bound testosterone was positively correlated with sexual desire and ED. Therefore, the reduced synthesis of albumin may affect erectile function. Long-term protein malnutrition in protein also leads to decreased muscle strength and hypoproteinemia, which is closely linked to ED. Platelets are recognized as key mediators of the immune system and thrombosis. Activated platelets generate chemokines and cytokines to enhance the recruitment of neutrophils. Meanwhile, an increase in the levels of inflammatory mediators damages vascular endothelial cells, forming a vicious cycle of platelet activation. Previous studies have identified a correlation between adverse clinical outcomes and platelet reactivity. Lymphocytes mediate adaptive immunity and play a decisive role in innate immunity. Collectively, the current study validated that hemoglobin, albumin platelets, and lymphocytes play vital roles in organismic nutrition and inflammation, which is related to erectile function.

ED is a complex multi-factorial disease associated with various risk factors, including depression, diabetes, hypertension, dyslipidemia and cardiovascular disease. However, to data, no study has explored the correlation between HALP and ED. This study included 906 ED participants and found that the ED group had a lower average HALP score than the control group. Thus, our results corroborate that nutrition and inflammation play a pivotal role in the physiopathology of ED. Penile erection relies on the physiological functioning of the endothelium, which produces nitric oxide to increase arterial flow^20^. Inflammation and oxidative stress can damage the vascular endothelium, thereby limiting NO production and restricting blood flow^21^. Previous studies have identified that the levels of inflammatory markers were abnormally expressed in ED patients. Liao et al. performed a comparative study involving 113 ED patients and uncovered that the neutrophil count, neutrophil–lymphocyte ratio, and platelet-lymphocyte ratio were higher in the ED group compared to the control group, whereas lymphocyte counts were lower^22^. Similarly, Ventimiglia et al. performed a real-life study involving 279 ED patients and determined that NLR > 3 may be an independent predictor of severe ED^23^. Zhang et al. performed a meta-analysis involving 7 relevant studies and identified NLR and PLR as independent risk factors for ED^8^. In addition, serum C-reactive protein-albumin ratio and serum high-sensitivity C-reactive protein have been associated with ED risk^7,24^. It is worthwhile emphasizing that PDE5i treatment can significantly reduce serum C-reactive protein levels^7^. Hence, HALP can reliably predict the risk of ED.

The present study highlights the relevance of immune and nutritional status in ED. In other words, improving systemic immunity and nutritional status might reduce the risk of ED. In addition, HALP might serve as an indicator for assessing the risk of ED. Nevertheless, some limitations of our study should not be overlooked. Firstly, this research was based on data collected from the NHANES database, which is limited to the US population. Consequently, our results may not be generalizable to the global population. Secondly, the diagnosis of ED was made based on self-reported responses to simple questionnaires. Using the International Erectile Function Index questionnaire may offer a more scientific approach to diagnosis of ED. Finally, data on covariates that may influence ED, such as dyslipidemia and depression, were not collected in this study.

## Conclusion

In summary, this cross-sectional study identified a non-linear correlation between HALP scores and the risk of ED. Patients with high levels of HALP had a significantly lower risk of ED than those with low HALP scores. The relationship was more prominent in participants aged ≥ 50 years, as well as those with hypertension and diabetes. However, HALP and its individual parameters may not mediate the association between urinary cobalt exposure and the risk of ED. Nonetheless, further studies are warranted to confirm our conclusion and explore underlying mechanisms.

## Data Availability

All data are publicly available from the NHANES.
